# Alone together online: a mixed-methods analysis of loneliness, recognition, and peer response in a public digital community

**DOI:** 10.3389/fpsyg.2026.1860516

**Published:** 2026-07-15

**Authors:** Anurag Shekhar, Calvin Mabaso

**Affiliations:** Department of Industrial Psychology and People Management, College of Business and Economics, University of Johannesburg, Johannesburg, South Africa

**Keywords:** digital community, gender, loneliness, mixed methods, peer support, recognition, Reddit, reflexive thematic analysis

## Abstract

**Introduction:**

Loneliness is a significant public health concern, yet most research relies on survey-based methods that capture prevalence and correlates rather than the experiential and social texture of how loneliness is described and responded to in everyday settings. This study examined loneliness-related discourse in r/lonely, one of the largest public subreddits dedicated to the topic.

**Methods:**

The study used a convergent mixed-methods design combining large-scale computational text analysis and Braun and Clarke reflexive thematic analysis. The computational strand analyzed 877,799 comments using sentiment analysis, emotion analysis, topic modeling, and keyword co-occurrence network analysis. The qualitative strand analyzed a purposive high-engagement sample of 80 posts and 478 associated comments for thread-based analysis in ATLAS.ti.

**Results:**

Five themes were developed: plural forms of loneliness; longing and accumulated disappointment; gendered loneliness and contested recognition; uneven peer support; and rejection of misrecognizing advice. Computational findings showed that positive sentiment and supportive emotional language were prominent at scale, but the qualitative analysis revealed substantial variation in response quality, including predatory behavior, gendered dismissal, and forms of misrecognition that compounded rather than addressed loneliness.

**Discussion:**

The findings contribute to understanding loneliness as a multidimensional, socially negotiated condition shaped by relational injury and the uneven politics of recognition in public digital communities.

## 1 Introduction

Loneliness has become an increasingly visible public health and social concern. In 2023, the U.S. Surgeon General described it as an epidemic, estimating that roughly half of American adults experience it to some degree (U.S. Public Health Service, 2023). Global meta-analyses suggest that about one in four older adults worldwide feels lonely, although the problem is not limited to later life (Hajek et al., 2025; Surkalim et al., 2022). Younger adults, especially those in their twenties, also report high levels of loneliness, and the COVID-19 pandemic appears to have intensified an already growing problem (Ernst et al., 2022). The effects extend beyond emotional discomfort. Chronic loneliness has been linked to depression, cardiovascular disease, cognitive decline, and premature mortality, with some work suggesting health risks comparable in scale to smoking (Hawkley and Cacioppo, 2010; Holt-Lunstad et al., 2015). Taken together, these findings position loneliness as a serious and persistent concern.

Loneliness is difficult to define precisely because it is not the same as being alone. A person may feel profoundly lonely while surrounded by others, in a relationship, or within a family, while another may spend much of life alone without experiencing that condition as painful (Luhmann et al., 2022; Mund et al., 2020). Weiss (1973) drew an influential distinction between emotional loneliness, which arises from the absence of an intimate attachment figure, and social loneliness, which arises from the absence of a broader social network. More recent scholarship has extended this understanding by pointing to the diversity of loneliness experiences and questioning whether standard measurement tools fully capture that heterogeneity (Maes et al., 2022; Van Tilburg, 2021; Walsh et al., 2025). Beneath these distinctions lies a broader claim made by Baumeister and Leary (1995): the need to belong is not merely a social preference but a fundamental human motivation, and its frustration carries serious psychological consequences. From this perspective, loneliness is not simply a passing mood. It is a signal that an important human need is unmet or insecure.

Despite growing research attention, the qualitative texture of loneliness remains less well understood than its prevalence and correlates. Large-scale studies have been valuable in documenting rates, risk factors, and health associations, but they are less well suited to capturing how loneliness is experienced, narrated, and interpreted in everyday life (Mansfield et al., 2021; McKenna-Plumley et al., 2020). Qualitative research has begun to address this gap, yet it often relies on purposively recruited samples, which can range from small interview cohorts (Birken et al., 2023) to larger, free-text survey datasets (Zheng et al., 2023). Those approaches are valuable, but they have a structural limitation that is relevant here. Interviews and open-ended questionnaires are researcher-initiated: the topics, the framing, and the moment of disclosure are all shaped by the research encounter itself. Andreotta et al. (2019) draw a direct contrast, noting that unlike survey or laboratory-based methods, social media data emerges from real-world social environments without any prompting from researchers, meaning participant behavior is relatively unconstrained by researcher influence. Salzmann-Erikson and Olsson (2025) make the same point specifically for Reddit, describing its user-generated narratives as ecologically valid and unsolicited expressions that approximate the lived experience of loneliness as articulated in everyday digital language, rather than in response to constrained survey formats. Sit et al. (2024) further note that pre-existing forum data reveals themes that may not be apparent when using traditional modes of data collection, because users are communicating in their own words without researcher prompts. Beyond the absence of reactivity, Reddit data also carry an interactional quality that interview methods cannot replicate: disclosure is followed by peer response in real time, from others with shared experience, without researcher mediation. That dynamic is central to the research questions this study addresses.

Three gaps in the existing literature motivate this study. Large-scale loneliness research has been valuable in establishing prevalence, risk factors, and health consequences, but survey-based methods are less suited to capturing how loneliness is described, narrated, and interpreted in people’s own words (Mansfield et al., 2021; McKenna-Plumley et al., 2020). Qualitative studies have begun to address this, but they typically rely on small recruited samples in interview or clinical settings, which do not capture how loneliness is expressed in naturally occurring peer interaction (Birken et al., 2023; Zheng et al., 2023). Perhaps most importantly, relatively little work examines loneliness as an interactional phenomenon—how disclosures of loneliness are received, validated, disputed, or dismissed by others in public digital settings. Understanding that dimension requires a setting where loneliness is expressed spontaneously and where responses from others are directly observable.

Online communities now provide one of the clearest settings in which such expressions can be observed. In online peer communities, this expression takes a specific form: users disclose their loneliness publicly, addressing an unknown audience and receiving responses from peers who may share similar experiences. Understanding how that disclosure is received (whether with empathy, dismissal, or something more harmful) is as important as understanding the loneliness itself. Digital platforms, and Reddit in particular, have become important spaces for disclosing loneliness, distress, and relational difficulty (De Choudhury and De, 2014; Salzmann-Erikson and Olsson, 2025). Reddit’s anonymity affordances, including pseudonyms, throwaway accounts, and community-specific forums, reduce some of the social costs of disclosure and create conditions in which users may share experiences they would not voice in identifiable settings (Amaya et al., 2021; Proferes et al., 2021). The public subreddit r/lonely, with its thread-based discussions and visible engagement patterns, offers a naturally occurring archive of loneliness disclosure and peer response at a scale that conventional recruitment-based studies are unlikely to achieve. It also brings into view questions that extend beyond the experience of loneliness itself. How is loneliness described? How do other users respond to it? What forms of support, validation, dismissal, or tension emerge in reply? These are relational questions, and they require a design that can capture both large-scale patterning and the meaning of individual exchanges. Gender is a well-established dimension of how loneliness is expressed and socially recognized. Research shows that gendered norms shape both the willingness to disclose loneliness and the way such disclosures are received by others, with men and women facing different barriers to having their experiences acknowledged (Rees et al., 2024; Wagner and Reifegerste, 2024; Walsh et al., 2025). Three research questions guide the study. First, what emotional, thematic, and lexical patterns characterize loneliness-related discussion in a large public online peer community? Second, how do users in high-engagement threads describe the forms, causes, and emotional consequences of loneliness? Third, how do other users respond to expressions of loneliness, and what do those responses reveal about recognition, peer support, and the social dynamics of a public digital community? The first question is addressed through large-scale computational analysis of the comment corpus. The second and third are addressed through reflexive thematic analysis of a high-engagement qualitative subsample, with the findings integrated in a dedicated mixed-methods section.

This study addresses those questions through a mixed-methods design that combines large-scale computational text analysis with Braun and Clarke reflexive thematic analysis of a high-engagement qualitative subsample (Braun and Clarke, 2019, 2021). The computational phase analyzed a corpus of 69,313 posts and 877,823 associated comments from r/lonely using sentiment analysis, NRC emotion analysis, topic modeling, and keyword co-occurrence network analysis. For the qualitative phase, the 80 most liked posts in the corpus were selected, together with their associated comment threads, producing a sample of 558 posts and comments with a combined engagement score of 85,411 likes. These were subjected to close reading and reflexive thematic analysis in ATLAS.ti. The two strands were designed to complement one another. The computational analyses identify what is prominent and recurring across the wider corpus, while the thematic analysis examines how those patterns are expressed, interpreted, and negotiated within particular threads.

The study is anchored theoretically in the need to belong perspective and draws additional support from Weiss’s distinction between emotional and social loneliness, rejection and social pain research, and recognition theory, particularly Honneth’s account of the harms associated with misrecognition. This combination is useful because the dataset is not only about the absence of connection. It is also about the unequal recognition of loneliness claims. Posts and comments in r/lonely do not simply describe isolation. They also show how loneliness is interpreted by others, how some disclosures are dismissed or sexualized, and how users respond when advice or support fails to recognize the nature of their distress.

The study makes three contributions. Theoretically, it extends recognition theory into the context of digital peer communities by showing that misrecognition operates not only in the social world from which users feel excluded, but within the support spaces they turn to as an alternative. This extends Honneth’s framework beyond interpersonal and institutional settings to online discourse environments where belonging is publicly negotiated. Second, the study advances understanding of loneliness as a socially contested rather than purely individual condition, demonstrating that how loneliness is received by others—including whether it is validated, dismissed, or gendered—shapes the experience of loneliness itself. This has implications for how intervention designers and community moderators conceptualize peer support: the response environment is part of the problem, not only part of the solution. Third, the study demonstrates a methodologically productive integration of computational and reflexive qualitative analysis for sensitive online discourse, showing that aggregate sentiment and lexical patterns require close interpretive analysis to avoid misleading conclusions about community wellbeing.

## 2 Literature review

### 2.1 Conceptualizing loneliness

Loneliness is generally defined as a subjective and distressing experience arising from a perceived gap between the relationships a person has and those they want (Perlman and Peplau, 1981). It is therefore distinct from solitude. Being alone is an objective condition, whereas loneliness is an emotional and evaluative response to one’s social world (Luhmann et al., 2022). The two do not necessarily coincide. People may feel lonely in the presence of others, and some may spend extended periods alone without experiencing that condition as painful (Mund et al., 2020; Ong et al., 2016).

A key distinction in the literature comes from Weiss (1973), who differentiated emotional loneliness from social loneliness. Emotional loneliness arises from the absence of an intimate attachment figure, whereas social loneliness arises from the lack of a satisfying network of peers. This distinction remains useful because it suggests that loneliness is not a single uniform state. More recent work has extended this view by proposing additional dimensions, including existential loneliness, and by questioning whether standard measurement tools fully capture the range of loneliness experiences people report (Maes et al., 2022; Van Tilburg, 2021; Walsh et al., 2025). This body of work supports the view that loneliness should be treated as multidimensional rather than reducible to a simple measure of social isolation.

### 2.2 Prevalence, causes, and consequences

Loneliness is widespread across age groups and regions, although its distribution is uneven. Meta-analytic evidence suggests that around one in four older adults worldwide experiences loneliness, while younger adults also report high levels, especially during periods of instability and transition (Ernst et al., 2022; Hajek et al., 2025; Surkalim et al., 2022). Public health concern has grown accordingly, with the U.S. Surgeon General’s 2023 advisory describing loneliness as a major contemporary challenge linked to broader patterns of social disconnection (U.S. Public Health Service, 2023). Recent evidence also suggests that digital technology is not straightforwardly a cause of loneliness. Hall (2025) found that social media use is weakly related to trait loneliness, explains little variance relative to other predictors, and does not predict change in loneliness over time, concluding there is no evidence it causes loneliness. During the COVID-19 pandemic, however, online communities took on greater significance as spaces for social support, with Bak et al. (2023) observing a shift in loneliness-related Reddit discussions from dating concerns to online interaction and community, suggesting that digital platforms can become more central to social life under conditions of broader social restriction.

The causes of loneliness are varied and often cumulative. Common triggers include bereavement, divorce, relocation, retirement, and disruptions to expected social continuity (Hajek et al., 2025; Hong et al., 2024). Relational injury is especially important. Research on rejection, exclusion, and ghosting suggests that social disconnection is often experienced as a form of interpersonal harm rather than simple absence (Cacioppo and Cacioppo, 2014; Pancani et al., 2022; Shapiro, 2024).

The consequences of persistent loneliness are well documented. They include increased risk of depression, anxiety, cardiovascular disease, dementia, and premature mortality (Hawkley and Cacioppo, 2010; Hong et al., 2024). Holt-Lunstad et al. (2015) found that the mortality risk associated with social disconnection is substantial and comparable to major behavioral health risks. Hawkley and Cacioppo (2010) further argued that chronic loneliness can become self-reinforcing, heightening vigilance for social threat, distorting social perception, and encouraging withdrawal. This literature establishes loneliness as both common and consequential. One clarification is important for the present study. This paper does not examine loneliness caused by social media use or technology dependence. It examines general loneliness (social, emotional, existential, and relational) as it is expressed and responded to within a public online community. Reddit is the setting in which that loneliness is disclosed and discussed, not its cause. Users bring loneliness shaped by offline relationships, life events, and relational injury into a digital space. The study is concerned with how that loneliness is narrated and socially received in that setting.

### 2.3 Gender and the social recognition of loneliness

In this study, gender refers to both the social norms and expectations that shape how loneliness is expressed and recognized, and the way biological sex categories are used by users themselves to frame, challenge, or dismiss loneliness claims within the community. The relationship between gender and loneliness is inconsistent in the literature. Some studies report higher loneliness among women, while others suggest that men and women may experience different forms of loneliness rather than different amounts, with men more commonly linked to social loneliness and women to emotional loneliness, particularly in later life (De Jong Gierveld and Havens, 2004; Hong et al., 2024; Walsh et al., 2025). These inconsistencies suggest that gender differences in loneliness are less about prevalence and more about how loneliness is shaped, expressed, and responded to within particular contexts.

Of direct relevance to this study is how gender structures the social recognition of loneliness claims. Wagner and Reifegerste (2024) found that loneliness and conformity to masculine norms together mediate men’s non-disclosure of mental distress, with the internalized archetype of the strong and silent man making emotional vulnerability appear unmanly and reinforcing patterns of silence. Rees et al. (2024) similarly found that men in later life frequently associated being busy with not being lonely, using the language of self-sufficiency to mask relational need. These patterns suggest that male loneliness often goes unrecognized not because it is less real, but because the social scripts available to men make it harder to name and share.

Women may face different barriers to recognition online; while platforms offer support, the lack of traditional social cues can lead to the dismissal of feelings if responses do not align with the specific relational deficit disclosed (Marshall et al., 2024; Salzmann-Erikson and Olsson, 2025). Gender is therefore relevant to this study at two levels: it shapes the subjective experience of loneliness, and it structures whether and how loneliness claims are taken seriously by others. Both dimensions are visible in the Reddit data and form a central part of the qualitative analysis.

### 2.4 Reddit as a site for loneliness disclosure and peer response

Digital platforms have become important spaces in which loneliness is expressed and managed. Reddit is particularly suited to this kind of research for several reasons that distinguish it from other online forums and social media platforms. Unlike Facebook or Instagram, which are organized around real-name identities and existing social networks, Reddit operates through pseudonymity and topic-based communities, allowing users to disclose sensitive experiences to an unknown public without linking those disclosures to their offline identity (Amaya et al., 2021; Proferes et al., 2021). This anonymity architecture is consequential: Kahlow (2024) found that Reddit facilitates deeper and more personal disclosures than identifiable platforms, specifically enabling what she terms core disclosures involving personal inadequacies, fears, and unmet needs. Unlike single-topic forums or chat-based platforms, Reddit’s thread structure preserves the full interactional sequence of a disclosure and its replies, making it possible to analyze not only what users say but how others respond. The upvote mechanism adds a further analytical dimension: it provides a community-generated signal of resonance and visibility that other forums lack, allowing researchers to identify which experiences and responses the community finds most significant (Leavitt and Robinson, 2017; Proferes et al., 2021). Taken together, these features make Reddit a distinctive and analytically rich source for studying how loneliness is expressed and socially received. Reddit is especially relevant because it combines pseudonymity, topic-based communities, and thread-based discussion, allowing users to disclose sensitive experiences to a broad but loosely bounded audience (Amaya et al., 2021; Proferes et al., 2021). Users are often addressing an unknown public rather than an existing personal network, which makes the platform especially useful for studying peer response to vulnerability.

Research suggests that anonymity can reduce the social cost of disclosure and enable people to discuss experiences they might avoid in identifiable settings (Andalibi et al., 2018; Kahlow, 2024; Triggs et al., 2021). Reddit has therefore become an important site for research on mental health, distress, and peer support (De Choudhury and De, 2014; Salzmann-Erikson and Olsson, 2025). At the same time, the benefits of online peer support are conditional. Supportive replies can validate experience and reduce immediate distress, but online spaces can also produce co-rumination, conflict, emotional contagion, or harmful interaction (Chen and Xu, 2021; Dana et al., 2024; Lavis and Winter, 2020; Mansfield et al., 2021; Marshall et al., 2024). These mixed possibilities make digital communities especially important for a study concerned not only with loneliness itself, but with how loneliness is received by others.

## 3 Theoretical framework

This study is anchored primarily in the need to belong perspective (Baumeister and Leary, 1995), which holds that human beings have a fundamental motivation to form and maintain stable, meaningful, and reciprocal social bonds. Baumeister and Leary argued that this need is not a preference but a basic drive, comparable in urgency to physiological needs, and that its frustration produces reliable and serious psychological consequences including anxiety, depression, and impaired cognitive functioning. Within this framework, loneliness is understood as a signal that the need to belong is unmet or under threat.

To capture the different forms loneliness can take, the study draws on Weiss’s (1973) distinction between emotional loneliness and social loneliness. Emotional loneliness arises from the absence of an intimate attachment figure and produces feelings of anxiety and emptiness. Social loneliness arises from the lack of a satisfying network of peers and produces feelings of boredom and marginality. Weiss argued that these two states require different remedies: social integration through friendships cannot compensate for the absence of intimate attachment, and vice versa. This distinction is theoretically important because it means that loneliness is not a single uniform condition and that different forms of relational deficit imply different needs.

A third supporting lens comes from rejection and social pain research. Cacioppo and Cacioppo (2014) proposed that perceived social isolation functions as a biological and psychological alarm system analogous to physical pain, signaling that an individual’s social connections are threatened or damaged. Being on the social perimeter triggers a self-preservation mode characterized by hypervigilance for social threat, cognitive biases toward negative social information, and behavioral withdrawal—a cycle that deepens isolation over time. Pancani et al. (2022) extended this framework to modern digital relational dissolution, arguing that practices such as ghosting constitute a specific form of ostracism that threatens the basic human needs for belonging, self-esteem, control, and meaningful existence. Ghosting is particularly harmful, they argue, because the sudden withdrawal of communication prevents the account-making process through which people interpret and recover from relational endings. Without explanation, victims are left in ambiguity that can induce self-blame and sustained psychological distress.

The framework also draws on recognition theory, specifically Honneth’s (1996) account of misrecognition as a form of social and psychological injury. For Honneth, recognition (being seen, valued, and taken seriously by others) is a precondition for psychological integrity. Misrecognition, which occurs when a person’s self-understanding or legitimate needs are denied, distorted, or dismissed by others, constitutes harm in its own right, independent of any material deprivation. This lens is relevant because loneliness in peer communities involves not only the absence of connection but the active social adjudication of whose loneliness counts and whose does not.

**FIGURE 1:**
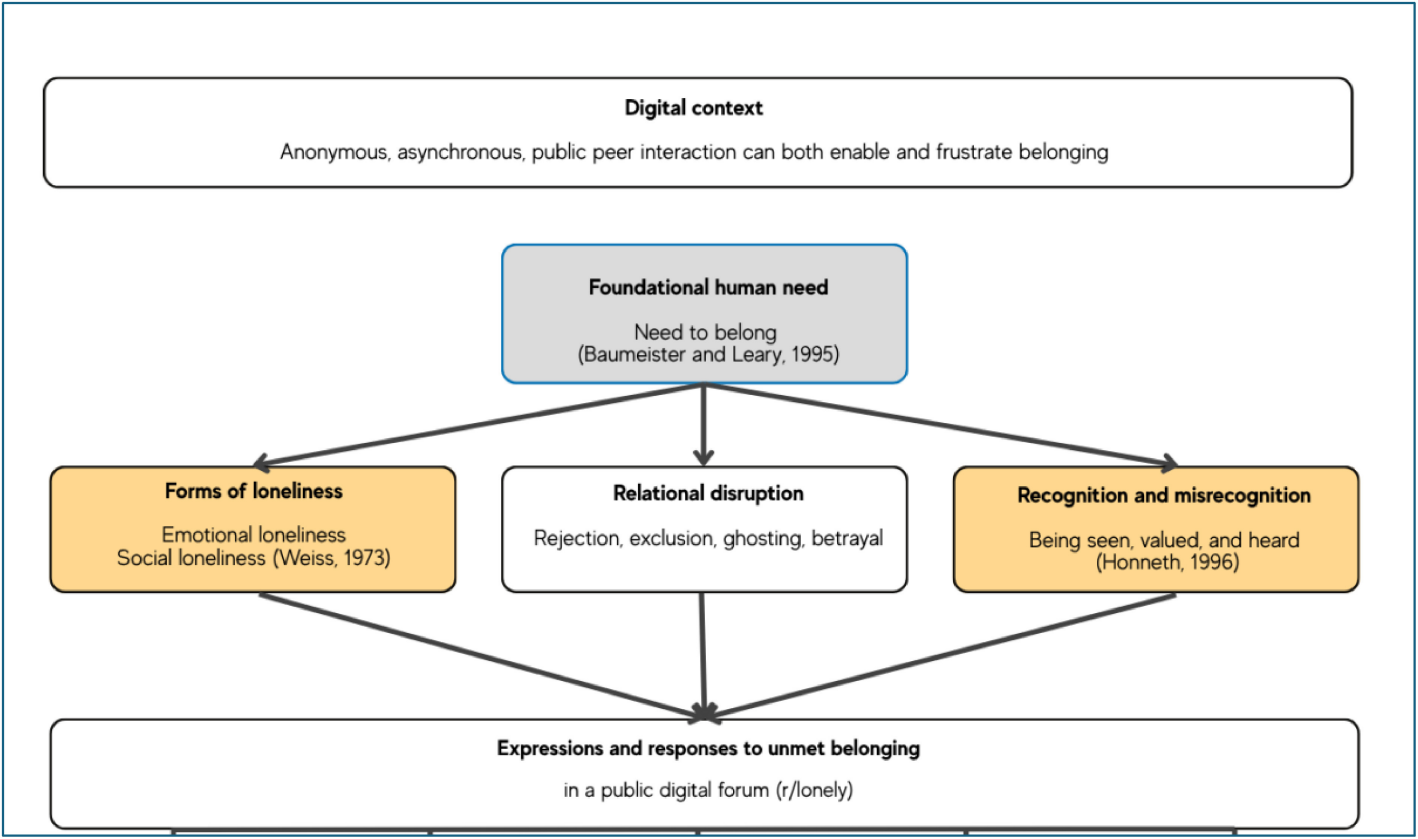
Theoretical framework for the r/lonely study. The framework positions the need to belong as the primary conceptual anchor, supported by Weiss’s distinction between emotional and social loneliness, rejection and social pain perspectives, and recognition theory. The three intermediate constructs converge on the analysis of expressions and responses to unmet belonging in a public digital forum.

These four perspectives are applied here to a public digital peer community, where anonymous, asynchronous interaction creates specific conditions for both disclosure and response (see Figure 1). The digital context shapes how belonging needs are pursued, how relational injury is experienced and narrated, and how recognition or misrecognition operates when loneliness claims enter a public space. Taken together, the framework provides a coherent basis for understanding loneliness as an unmet need for belonging, expressed in different forms, intensified by rejection and social injury, and negotiated through the unequal recognition of claims in a public online forum.

## 4 Methodology

### 4.1 Research design

This study used a convergent mixed-methods design, in which computational and qualitative analyses were conducted in parallel and brought together during interpretation (Creswell and Plano Clark, 2017). This design was chosen because the research questions required both breadth and depth. The computational strand was used to examine the larger Reddit corpus at scale, identifying broad emotional, thematic, and lexical patterns that would not be visible through close reading alone. The qualitative strand was used to examine how loneliness was described, interpreted, and responded to within specific threads. Each strand addressed a different aspect of the dataset, and neither would have been sufficient on its own.

The choice of design also reflects the nature of the data. Discussions in r/lonely are large in volume, emotionally varied, and interactional in form. A purely computational approach could identify aggregate patterns, but it could not explain how users made sense of loneliness in context or how replies shaped the meaning of those disclosures. A purely qualitative approach would allow for interpretive depth, but it would not capture the broader structure of the corpus or show whether the selected threads reflected wider patterns in the subreddit. The mixed-methods design therefore made it possible to analyze loneliness both as a large-scale pattern of online expression and as a lived, socially negotiated experience within particular exchanges.

### 4.2 Data collection

Data were obtained from the public r/lonely subreddit. This subreddit is a public online community in which users discuss loneliness, social isolation, rejection, relationship difficulties, and problems with belonging. Because the study aimed to examine recent patterns of interaction, the full dataset was first inspected and then restricted to posts created between 15 March 2023 and 15 March 2026.

The initial corpus contained 492,067 submissions and 2,869,437 comments. Restricting the dataset to the last 3 years reduced this to 235,547 submissions and 1,372,967 comments. To reduce low-engagement material while retaining a large and active corpus, alternative score thresholds from 1 to 5 were compared. A minimum score of 3 was selected because it provided a reasonable balance between scale and selectivity. Applying this threshold produced a final analytic corpus of 69,313 submissions and 877,823 comments attached to those submissions, giving a combined dataset of 947,136 text items.

This filtering strategy served two purposes. Limiting the corpus to recent years kept the analysis focused on current patterns of loneliness-related discussion. The score threshold reduced the influence of posts that attracted little visible engagement. All comments attached to the selected submissions were retained so that replies could be interpreted in relation to the posts that prompted them, rather than as isolated remarks.

Usernames and other directly identifying information were removed before analysis. Like counts were retained as indicators of visible community engagement. These were treated as indicators of visibility and community engagement within the subreddit, rather than as direct measures of importance, truth, or psychological intensity.

For the qualitative phase, a purposive high-engagement subsample was drawn from the 80 most liked posts in the final corpus and 478 associated comments, producing a total qualitative dataset of 558 text items. Comments were selected from among the most liked responses attached to those posts. The number of 80 posts was determined on pragmatic and analytic grounds. It was sufficient to generate thematic saturation across a range of loneliness experiences and response types while remaining feasible for detailed thread-based coding in ATLAS.ti. In reflexive thematic analysis, sample size is guided by the richness and diversity of the data rather than by statistical power requirements (Braun and Clarke, 2021). This purposive high-engagement sampling strategy was chosen because highly liked posts represent the forms of loneliness expression that achieved the greatest visibility and community resonance within the subreddit, making them analytically appropriate for examining how loneliness is publicly expressed and responded to. It was not intended to capture the full range of individual experiences across the subreddit.

This approach produced a manageable thread-based dataset for ATLAS.ti coding while preserving the interactional context of each discussion.

This sampling strategy also has limits. A high-engagement subsample favors posts and comments that were more visible, more resonant, or more readily engaged with by other users. Less visible contributions, low-engagement posts, and the experiences of users who did not post are not captured in the same way. The qualitative findings should therefore be read as an analysis of the forms of loneliness and response that received the greatest visibility within this subreddit, rather than as a full account of all experiences present in the broader population.

### 4.3 Computational analysis

Four main computational techniques were applied to the quantitative corpus.

#### 4.3.1 Sentiment analysis

Sentiment analysis was conducted using the Valence Aware Dictionary and Sentiment Reasoner (VADER), a lexicon-based tool developed for informal social media text, including material containing slang, emoticons, and non-standard punctuation (Hutto and Gilbert, 2014). Each comment received a compound score ranging from -1 to +1. Standard classification thresholds were applied: positive (≥0.05), neutral (between –0.05 and 0.05), and negative (≤–0.05). This analysis was used to provide a broad polarity profile of the comment corpus.

#### 4.3.2 Emotion analysis

Emotion analysis used the NRC Emotion Lexicon (Mohammad and Turney, 2013), which maps words onto eight emotion categories: anger, fear, anticipation, trust, surprise, sadness, joy, and disgust (Mohammad and Turney, 2013; Plutchik, 1980), as well as positive and negative sentiment polarities. Lexicon matches were calculated across the corpus to produce an emotional profile that was more differentiated than sentiment classification alone. In this study, the NRC analysis was used to identify the relative prominence of different emotional categories across the comment corpus.

VADER and the NRC Emotion Lexicon were selected because they are transparent, widely used, and well suited to the descriptive aims of large-scale social media analysis (Ribeiro et al., 2016). In the present study, their role was not to infer deep psychological states from individual users, but to characterize broad emotional tendencies across the corpus in a way that could later be interpreted alongside the qualitative findings (Thelwall, 2016).

#### 4.3.3 Topic modeling

Topic modeling was conducted using non-negative matrix factorization (NMF). NMF decomposes a document-term matrix by constraining all values to be non-negative, which tends to produce additive, parts-based representations that are often more interpretable than probabilistic alternatives when applied to short social media texts (Andreotta et al., 2019; Lee and Seung, 1999; O’Callaghan et al., 2015). In this study, topic modeling was used as an exploratory step to identify recurring lexical clusters in the comment corpus. To keep the analysis computationally manageable, the model was estimated on a capped subset of comments. After stopword removal and text cleaning, 48,083 comments were retained for topic modeling. Ten topics were retained following iterative testing for coherence and interpretability (Röder et al., 2015). These outputs were treated as exploratory groupings of terms rather than as final thematic categories or statistically generalizable representations of the full corpus.

#### 4.3.4 Keyword co-occurrence network analysis

Keyword co-occurrence network analysis was used to examine how frequently recurring terms were connected across the corpus (Callon et al., 1983). To keep the analysis computationally manageable, it was conducted on a capped sample of 300,000 comments drawn from the main dataset. In the resulting network, nodes represented retained keywords and edges represented their co-occurrence within the same comment. Edge weight indicated the frequency of co-occurrence (Van Eck and Waltman, 2010), and node centrality was assessed through weighted degree and document frequency (Newman, 2004). This analysis was used to identify broad lexical structure and highly connected terms within a large subset of the corpus. The findings are interpreted as exploratory and descriptive rather than as generalizable to every comment in the full dataset.

In addition to these four main techniques, lexical frequency checks were used during data preparation and interpretation to inspect recurring single words and short phrases. These checks informed stopword refinement, helped identify residual platform artifacts, and supported interpretation of the topic and keyword analyses. They were treated as supplementary rather than standalone analytic outputs.

### 4.4 Reflexive thematic analysis

The qualitative subsample was analyzed using Braun and Clarke’s reflexive thematic analysis (Braun and Clarke, 2019, 2021). This approach was selected because it is well suited to the interpretive analysis of online discourse and to research concerned with patterns of shared meaning rather than with coding consensus alone. In this study, themes were understood as the outcome of sustained analytical engagement with the data, not as fixed entities waiting to be discovered. Reflexive thematic analysis accommodates both inductive and theoretically informed approaches (Braun and Clarke, 2019, 2021). In this study, the theoretical framework informed the analytic sensibility (directing attention toward forms of loneliness, relational injury, and recognition) but did not predetermine the themes. Codes were generated through close engagement with the data, and themes were developed iteratively across multiple rounds of analysis. The process was therefore neither purely inductive nor deductively driven by a fixed coding framework imposed prior to data engagement.

Analysis followed Braun and Clarke’s six phases: data familiarization, coding, initial theme development, theme review, theme definition and naming, and report writing (Braun and Clarke, 2019, 2021). The sampled posts and comments were read repeatedly to develop familiarity with both the content of individual threads and the broader patterns across them. Initial coding was conducted in ATLAS.ti, using close engagement with the text to identify recurring meanings related to the forms of loneliness described, their perceived causes, their emotional consequences, coping efforts, and the nature of responses from other users. Codes were then reviewed, grouped, and refined across multiple rounds of analysis.

Theme development focused on identifying coherent patterns of shared meaning across the dataset rather than simply collating frequently occurring codes. Attention was given to both semantic and more interpretive patterns in the material, while keeping the analysis grounded in the wording and interactional context of the threads. Reflexive memoing and analytic note-taking were used throughout to document interpretive decisions, emerging connections, and points of uncertainty. Primary coding and theme development were conducted by the first author, with ongoing reflexive discussion and interpretive review involving the second researcher.

Following Braun and Clarke’s position, this study did not use inter-rater reliability as a marker of quality, because reflexive thematic analysis does not treat themes as fixed data properties that can be independently verified in a positivist sense (Braun and Clarke, 2019, 2021). Rigor was supported instead through careful familiarization, iterative coding, transparent decision-making, reflexive memoing, and the development of themes that were grounded in multiple extracts across the corpus.

### 4.5 Mixed-methods integration

The two strands were integrated at the stage of meta-inference through systematic comparison and joint interpretation (Creswell and Plano Clark, 2017; Tashakkori et al., 2020). Consistent with the convergent design described in Section 5.1, each strand was conducted independently before the findings were brought together. Integration examined where the two strands converged, where one extended the interpretation of the other, and where the qualitative findings added explanatory depth to patterns identified computationally (Bryman, 2006; Greene et al., 1989).

At the first level, the computational analyses provided a macro-level account of the dataset (Morgan, 1998). Sentiment analysis, emotion analysis, topic modeling, and keyword network analysis established the broader emotional profile of the corpus and identified recurring areas of discussion, including relationships, work, social life, family context, and meaningful personal occasions. These outputs helped situate the qualitative sample within the wider structure of the subreddit.

At the second level, the qualitative findings were used to interpret patterns that the computational analyses could identify but not fully explain. For example, the quantitative results showed that supportive and distress-related language coexisted across the corpus, but they could not on their own explain how this coexistence was produced in interaction. The thematic analysis clarified that users often disclosed loneliness in terms of rejection, disappointment, and emotional pain, while replies frequently offered sympathy, validation, encouragement, or, in some cases, dismissal and tension. Integration therefore allowed the study to move beyond description of large-scale patterns and toward a more context-sensitive account of how loneliness was expressed and negotiated within the subreddit.

### 4.6 Researcher positionality

Reflexive qualitative research requires explicit attention to how researchers’ social locations, professional backgrounds, and interpretive perspectives may shape analysis (Braun and Clarke, 2019, 2021). The qualitative analysis was conducted by the first author, a South Asian male researcher in his early forties with more than 14 years of senior human resource leadership experience across South Africa and India, and doctoral training in Human Resource Development with a focus on wellbeing and mental health. The analysis was reviewed through ongoing reflexive discussion with a second researcher, an African university professor with extensive academic and research experience. Both researchers approached the dataset as analytical outsiders to the subreddit and had no personal involvement in online loneliness communities.

Throughout the analytic process, attention was given to how these professional and social positions might shape interpretation, particularly in relation to loneliness, gender, support, and online interaction. Reflexive memoing and discussion were used to document emerging interpretations, surface assumptions, and refine theme development. This process did not remove subjectivity from the analysis, but it helped make the interpretive process more transparent and considered.

### 4.7 Ethical considerations

This study analyzed publicly accessible Reddit content from a public subreddit and did not involve direct interaction with users, intervention, or the collection of directly identifying personal information. The South African Ethics in Health Research Guidelines state that research using information that is publicly available and does not require gatekeeping, site or platform permission may not require formal ethics review, depending on the ethical considerations relevant to the study (Department of Health Republic of South Africa, 2024). The guidelines further indicate that research involving observation in public spaces, including virtual public spaces, usually need not undergo formal ethics review where the researcher does not directly interact with individuals or groups, does not stage an intervention, participants do not have a reasonable expectation of privacy, and dissemination of findings does not identify individuals or groups. These conditions were met in the present study. The research relied on publicly accessible Reddit material, involved no contact with users, did not alter the online environment, and reported findings in a way that avoided identifying individuals.

At the same time, the topic of loneliness is sensitive, and public accessibility does not remove the need for careful ethical judgment. The study was therefore conducted in a way that minimized potential harm. No usernames or direct identifiers were retained in the analytic dataset reported in the manuscript. Quotations are presented without attribution and are used only to illustrate broader analytic patterns. Care was taken to reduce the risk of reverse identification and to handle the material proportionately to the sensitivity of the subject matter. In this sense, the study followed a cautious, context-sensitive approach to the ethical use of public online data.

## 5 Results

### 5.1 Computational findings

A total of 877,799 Reddit comments were analyzed computationally using four complementary techniques: sentiment analysis, emotion analysis, topic modeling, and keyword network analysis. These analyses were used to characterize the comment corpus at scale and to provide a broad quantitative overview of recurring emotional tones, themes, and linguistic patterns. They were intended to support, rather than replace, the qualitative analysis. The thematic interpretation remains grounded in close reading of selected threads, while the computational findings help situate those patterns within the wider corpus.

#### 5.1.1 Sentiment distribution

Sentiment analysis was applied to the comment corpus only; submission texts were not included in this analysis. Of the 877,799 non-empty comments analyzed, 486,692 (55.44%) were classified as positive, 214,141 (24.40%) as negative, and 176,966 (20.16%) as neutral (Table 1). The corpus leaned positive overall, though negative and neutral comments together accounted for nearly half the total. This distribution suggests that the comment space was not shaped solely by distress. At the same time, the positive majority should be interpreted with caution: VADER is a rule-based lexical tool that does not detect irony, sarcasm, or repetition, and emoji-heavy or sarcastically positive comments may be misclassified. The aggregate pattern is best understood as indicating that positive and negative interactional tones co-exist in this forum rather than as a precise characterization of comment content.

**TABLE 1:** Sentiment distribution of Reddit comments in the r/lonely corpus.

Sentiment	*n*	%
Positive	486,692	55.44
Negative	214,141	24.40
Neutral	176,966	20.16
Total	877,799	100.00

#### 5.1.2 Emotional profile

Emotion analysis using the NRC Emotion Lexicon was applied to the same comment corpus of 877,799 non-empty comments. Across all categories, the lexicon produced 7,347,039 emotion hits, and 635,961 comments contained at least one NRC emotion term. As shown in Table 2, positive emotion had the highest total hit count (1,535,344) and the widest comment coverage, appearing in 513,595 comments (58.51% of the corpus). Trust followed, appearing in 414,063 comments (47.17%), then negative emotion (382,490; 43.57%), joy (373,196; 42.51%), and anticipation (368,166; 41.94%). Sadness and fear each appeared in roughly one-third of comments (33.61 and 32.17%, respectively), while anger, disgust, and surprise were less widespread but still substantial. Disgust produced more total hits than surprise, though surprise appeared in slightly more comments, indicating that some categories were distributed more widely while others were more concentrated within a smaller set of texts.

**TABLE 2:** Emotion distribution of Reddit comments based on the NRC emotion lexicon.

Emotion	Total hits	Comments with emotion	Comment coverage %	Avg hits per comment
Positive	1,535,344	513,595	58.51	1.7491
Trust	934,103	414,063	47.17	1.0641
Negative	925,448	382,490	43.57	1.0543
Joy	811,418	373,196	42.51	0.9244
Anticipation	806,694	368,166	41.94	0.9190
Sadness	587,856	295,011	33.61	0.6697
Fear	542,649	282,367	32.17	0.6182
Anger	443,922	252,276	28.74	0.5057
Disgust	384,539	228,735	26.06	0.4381
Surprise	375,066	240,871	27.44	0.4273

Total hits refers to the number of NRC lexicon matches across the full corpus. Comment coverage refers to the percentage of comments containing at least one lexical match for a given emotion category. Average hits per comment was calculated using the full set of 877,799 non-empty comments analyzed. The NRC analysis covered all 10 emotion and sentiment categories.

The most notable feature of this distribution is that both supportive and distress-related emotional language appeared at scale across the same corpus. Positive and trust-related terms were the most common, but negative emotion, sadness, and fear were also prominent. This is consistent with a forum in which users disclose pain and isolation while replies often include reassurance or support. These findings describe the aggregate emotional register of the corpus rather than the emotional content of individual exchanges, since the NRC approach identifies lexical matches and cannot capture within-comment emotional complexity.

#### 5.1.3 Topic modeling

Topic modeling was used as an exploratory step to identify recurring lexical clusters in the comment corpus. To keep the analysis computationally manageable, the model was estimated on a capped subset of comments. After preprocessing, 48,083 comments were retained for topic modeling. Ten topic clusters were generated using non-negative matrix factorization (see Table 3). These clusters should be treated as broad lexical groupings rather than qualitative themes, and the findings are interpreted accordingly.

**TABLE 3:** Exploratory topic clusters identified through non-negative matrix factorization.

Topic	Top terms	Interpretive label
1	Deleted, ago, post, account, socials, says, knew, did, past, mental health	Deleted accounts, past events, and residual personal context
2	Birthday, happy birthday, happy, birthday hope, hope, enjoy, birthday friend, cake, wish, great	Birthday wishes and celebratory support
3	Removed, reddit, shame, completely, ve, sorry wish, mods, aspect, asf, reported	Moderation, removal, and platform artifacts
4	Thank, appreciate, thank appreciate, kind, thank kind, thank hope, hope, words, great, kind words	Gratitude and appreciative response
5	Don, think, friends, lonely, women, try, better, sure, right, men	Loneliness, friendships, gender, and general advice
6	Thanks, thanks man, man, appreciate, buddy, nice, hope, sure, try, thanks lovely	Informal appreciation and supportive exchange
7	Talk, hey, wanna, wanna talk, chat, friend, free, message, don talk, open	Offers to talk and invitations to connect
8	Good, luck, good luck, hope, night, good night, hope good, great, good idea, good morning	Encouragement, goodwill, and everyday care
9	Sorry, hear, hope, sorry hear, better, hope better, happened, sorry happened, man, soon	Sympathy, concern, and recovery wishes
10	Love, love love, hugs, chat, hey, send, hug, relationship, love chat, true	Affection, comfort, and emotionally warm contact

Topic labels were assigned by the researcher based on the highest-loading terms. The model was estimated on 48,083 pre-processed comments drawn from the main corpus. Topic modeling results are treated here as exploratory lexical clusters that support interpretation of the broader dataset. They are not presented as final qualitative themes.

Several clusters reflected interpersonal support and everyday peer response. Terms such as “thank,” “appreciate,” “kind words,” “sorry to hear,” “hope better,” “good luck,” and “happy birthday” appeared across multiple topics, indicating that these forms of expression were common in the comment space. Two clusters in particular (topics 4 and 6) both centered on gratitude and appreciation, suggesting that this was a particularly dense lexical area in the corpus. A further cluster (topic 7) organized around connection-seeking, with terms including “talk,” “chat,” “wanna talk,” “message,” and “open.” One broader cluster (topic 5) combined terms such as “friends,” “lonely,” “women,” “men,” and “try,” which appears to capture general discussion of loneliness, social relationships, and advice-giving.

Two clusters (topics 1 and 3) were dominated by platform-specific strings: “deleted” and “removed.” These almost certainly reflect Reddit’s literal account deletion and moderation removal labels rather than organic comment content. Their presence indicates that residual platform artifacts remain in the corpus despite preprocessing and should be read as data quality markers rather than substantively meaningful topics. Overall, the model suggests that the comment space was organized less around sharply separated issues and more around recurring registers of support, sympathy, encouragement, and attempts at connection.

#### 5.1.4 Keyword network analysis

Keyword co-occurrence network analysis was used as an exploratory technique to identify the most centrally connected terms across the comment corpus. The network was estimated on a capped sample of 300,000 comments. Terms were retained if they appeared at least 80 times and co-occurrence edges were retained if they appeared at least 30 times, producing a final network of 1,214 nodes and 11,243 edges.

**FIGURE 2:**
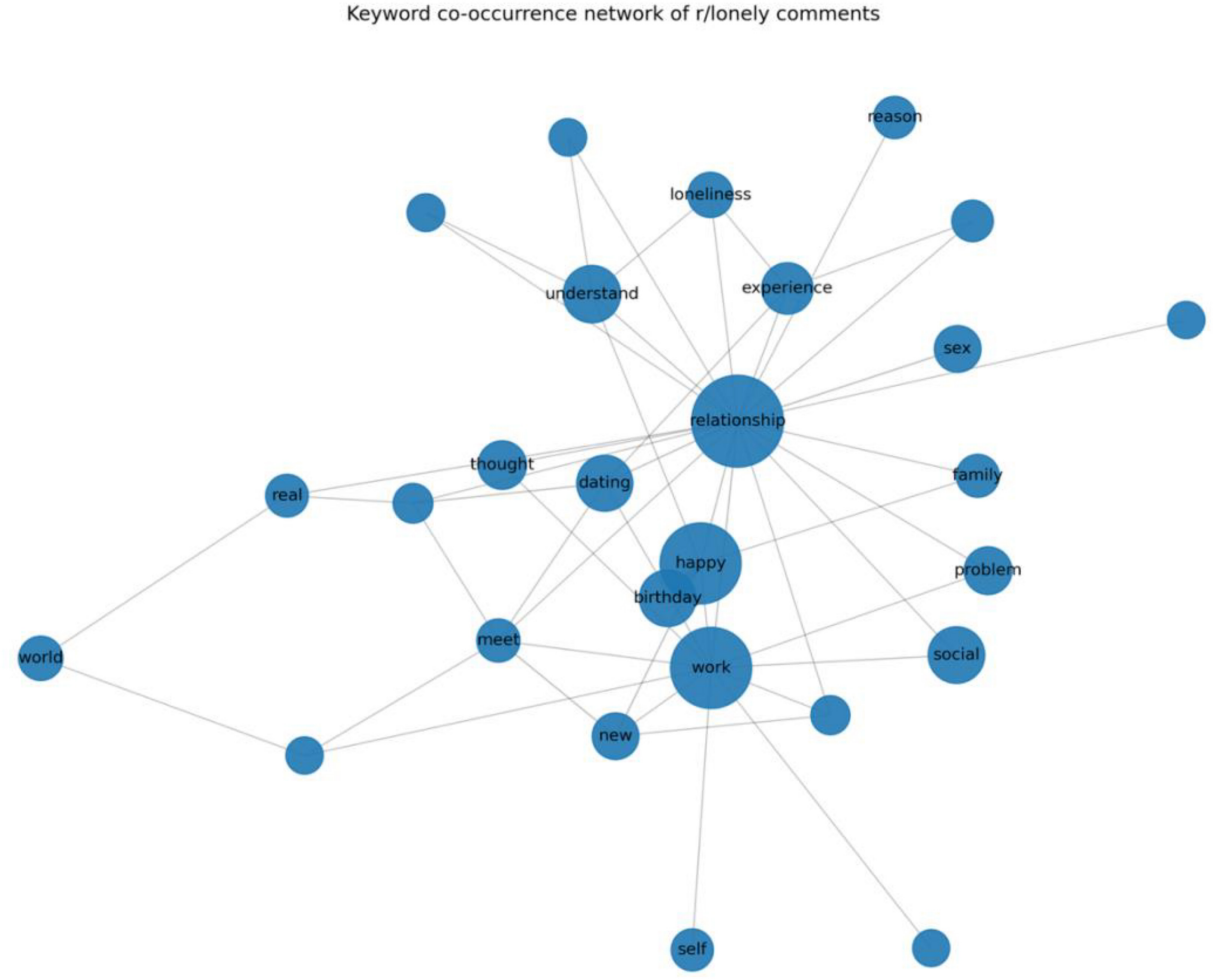
Simplified keyword co-occurrence network of Reddit comments from the r/lonely corpus after strict preprocessing. The figure displays the most strongly connected keywords in the analyzed sample. Node size reflects weighted degree, and edge thickness reflects co-occurrence strength between keyword pairs.

As shown in Figure 2 and Table 4, the most central keyword by weighted degree was *relationship*, followed by *work*, *happy*, *understand*, *birthday*, and *social* (the latter two sharing an identical weighted degree of 17,621). Further prominent terms included *dating*, *experience*, *problem*, *loneliness*, *family*, *online*, and *change*. This pattern points to several recurring areas of discussion: intimate and romantic relationships, employment and everyday routine, social interaction, family context, and attempts to situate loneliness within practical life circumstances. The presence of *birthday* as a highly connected term supports both the topic modeling findings and the qualitative observation that personally significant occasions frequently served as focal points for disclosure and peer response.

**TABLE 4:** Most central keywords in the Reddit comment network after strict preprocessing.

Keyword	Weighted degree	Document frequency	Interpretive relevance
Relationship	42,078	8,847	Intimate and relational concerns
Work	33,387	8,390	Employment, routine, and strain
Happy	33,323	13,875	Positive affect and supportive response
Understand	18,075	5,639	Validation and shared experience
Birthday	17,621	9,502	Personal milestones and social acknowledgment
Social	17,621	4,742	Social life and interaction
Dating	17,341	4,684	Romantic difficulty and partner-seeking
Experience	15,012	4,580	Lived experience and personal reflection
Thought	13,671	4,720	Self-reflection and interpretation
Problem	13,329	4,624	Perceived difficulties and obstacles
Sex	12,871	4,365	Intimacy and sexual concerns
New	12,845	4,578	Change, novelty, and new situations
Loneliness	12,228	4,190	Direct discussion of loneliness
World	11,870	4,315	Broader social or existential framing
Talking	11,861	4,330	Communication and interaction
Meet	11,393	3,789	Attempts to connect with others
Family	11,305	3,798	Family context and relational background
Real	11,202	4,592	Reality, authenticity, and self-positioning
Reason	11,031	4,129	Explanatory framing and sense-making
Self	10,964	3,685	Self-concept and self-evaluation

Weighted degree refers to the total strength of a keyword’s connections to other keywords in the co-occurrence network. Document frequency refers to the number of comments in which the keyword appeared within the analyzed sample of 300,000 comments. Interpretive labels were assigned by the researcher to summarize the broad area of meaning suggested by each term. The network results are treated as exploratory and descriptive rather than as final thematic findings.

The network does not indicate a single dominant concern. Some terms point to interpersonal difficulty, including *relationship*, *dating*, *sex*, *meet*, *ugly*, and *self*. Others suggest broader social or existential framing, such as *world*, *real*, *reason*, and *change*. Terms such as *work*, *family*, *social*, and *online* indicate that users often described loneliness in relation to ordinary settings and routines rather than as an isolated internal state. These are lexical co-occurrence patterns rather than meaning claims, however. The network shows which terms tend to appear together but cannot reveal the significance of those connections in context. For that reason, the network results are best read as a structural map of recurring language that grounds and complements the qualitative analysis.

Figure 2 shows a simplified keyword co-occurrence network derived from the strictly cleaned comment corpus. The visual pattern supports the results presented in Table 4 by showing how central terms were linked within the broader comment environment. Relationship is the dominant node and is connected to dating, sex, family, experience, and loneliness, suggesting that intimate and relational concerns were an important part of the corpus. Work forms another prominent node, while terms such as social, birthday, understand, and meet indicate that loneliness was also discussed in relation to everyday social life, personal milestones, and attempts to connect with others. The figure should be read as a selective visual summary of the strongest lexical connections rather than as an exhaustive representation of all keyword relationships in the dataset.

### 5.2 Qualitative findings: reflexive thematic analysis

The following five themes were developed through reflexive analysis of a qualitative sample drawn from 80 highly liked posts and 478 associated comments in r/lonely. The analysis addresses both how loneliness is described and how it is handled within a public peer-support forum. This is therefore not only an account of loneliness as a personal experience, but also of how that experience is recognized, challenged, responded to, and sometimes worsened in an online setting. The themes reflect recurring patterns of shared meaning rather than a full summary of everything present in the dataset.

#### 5.2.1 Theme 1: multiple forms of loneliness

Loneliness in this dataset does not appear as a single condition. Users describe social isolation, romantic loneliness, emotional loneliness, existential loneliness, and bodily loneliness. Seasonal and milestone-based loneliness also recur, especially around birthdays, Christmas, and New Year. Posts such as *“Today is my birthday. No one remembered. Not a single person”* and *“Alone—Pls Wish Me a Merry Christmas”* drew high engagement, suggesting that these socially significant moments made isolation feel especially visible.

What matters is not only that loneliness appears in different forms, but that users treat these forms as qualitatively different. Some describe being surrounded by people while still feeling profoundly disconnected. Others describe very small gestures of recognition as emotionally charged because more meaningful connection is absent. One post captures this clearly: *“I’m so lonely that I smile after i see someone upvotes me.”* Another asks, *“Do you ever feel so alone that the moment someone … treats you like a human being, you fall in love with them right away only to later realize how pitiful you really are?”* In both cases, ordinary acknowledgment carries disproportionate emotional weight because chronic loneliness has lowered the threshold of what counts as contact.

A distinctive sub-form within this theme is bodily loneliness, often named directly as touch starvation. These accounts describe the absence of physical contact as its own kind of deprivation. Several highly engaged posts describe incidental touch during surgery or dental treatment as unexpectedly profound. One user writes, *“I felt like I was human again for a moment,”* after being hugged by a coworker. Another describes hugging a plush toy at night while *“tearing up,”* using humor to soften what is, in effect, a disclosure of physical deprivation. These accounts suggest that prolonged absence of touch can erode not only comfort but a person’s sense of social existence.

A second form is existential emptiness. These posts move beyond loneliness as immediate pain and describe something closer to numbness or chronic inner vacancy. One user writes, *“It’s not about being lonely, it’s all about feeling empty chronically,”* before asking whether this has become part of who they are. A third form appears in overtly social settings such as school, parties, and birthdays, where exclusion becomes visible against the expectation of participation. These are not just accounts of being alone. They are accounts of being set apart in situations built for belonging.

Across these posts, loneliness is not static. It changes form, sharpens in particular contexts, and, over time, can alter how users experience themselves and the social world.

#### 5.2.2 Theme 2: longing and accumulated disappointment

Loneliness in this dataset is rarely described as passive. Users repeatedly describe trying to connect and failing. What defines this theme is not simply desire for closeness, but the exhaustion that follows repeated disappointment.

This is especially clear in posts where users recount effort in painful detail. One highly engaged post describes preparing carefully for a college speed dating event, only to see *“every woman’s smile start to fade”* when they sat down. Another describes being ghosted after date after date and concludes, *“I can’t imagine going through this all over again.”* In both cases, the emotional force lies not only in rejection itself, but in the hope and preparation that came before it. These are not users who never tried. They are users who feel worn down by trying.

Several posts identify physical appearance as a recurring source of rejection. One user writes, *“I’ve been called ugly all my life. It hurts every single time.”* Another asks whether it is *“a sin to be ugly,”* linking birthday abandonment to the feeling that their appearance makes people stay away. These accounts frame loneliness not just as bad luck, but as something structurally reinforced through repeated social experience.

That cumulative logic is named directly in one comment: *“The curse of loneliness is the longer you’re lonely, the more you crave love … The more desperate you seem, the less desirable you are … The less desirable you are, the lonelier you become.”* What matters here is not whether this account is objectively true, but that users experience loneliness as a self-reinforcing loop. Longing for connection remains present, but repeated failure changes its tone. It becomes guarded, bitter, or resigned.

Not all longing in this theme is romantic. Some of the most painful posts concern the loss of a specific person. A post about a partner’s suicide describes grief and loneliness as inseparable. This is not the loneliness of never having connected, but of having known connection and losing it. Across the theme, longing is specific, historically shaped, and worn down by experience.

#### 5.2.3 Theme 3: gendered loneliness and contested recognition

Gender is not just a background characteristic in this dataset. It is an explicit framework through which loneliness is interpreted, questioned, and at times denied. What makes this theme especially important is that it appears not only in original posts but in the replies, where users argue about whose loneliness is real, whose is worse, and what kind of experience should count.

Male loneliness is often described as being recast as sexual frustration rather than recognized as genuine emotional or relational deprivation. One highly engaged post states, *“Hate being a guy and saying I feel lonely. Everyone assumes its just sexual. I’m lonely in every aspect of the word.”* A reply captures the same frustration in simpler terms: *“being lonely is more than being horny.”* The complaint here is not only about feeling alone, but about having that feeling socially reduced to something else.

Women in the dataset describe a different but equally isolating problem. Several posters argue that male attention is routinely mistaken for meaningful connection. One user writes that repeated sexual messages *“don’t feel like real human interactions”* and make her feel like *“a sex object rather than a human being.”* Another sums up the difference bluntly: men may receive no response at all, while women may receive plenty of response that is exploitative, sexualized, or dehumanizing. The dataset repeatedly challenges the assumption that attention protects women from loneliness.

Some of the most striking comments in this theme reject simple comparisons altogether. Posts comparing men *“looking for water in a desert”* and women *“looking for clean water in a swamp”* or *“panning for gold in a sewer”* argue that the problem is not identical experience, but different forms of deprivation and risk. Other users reject the competitive framing itself, insisting that *“loneliness comes in every form imaginable”* and that the point should be mutual recognition rather than comparison. That such interventions are needed at all shows how strongly gender structures the way loneliness is interpreted within the subreddit.

#### 5.2.4 Theme 4: uneven peer support

The subreddit often functions as a meaningful source of peer support, but that support is neither stable nor evenly distributed. What emerges is a space where genuine care exists alongside dismissal, exploitation, and silence.

The strongest evidence of support comes from posts where users describe the subreddit as genuinely consequential. One writes, *“you guys saved my life,”* after receiving responses to a suicidal post and then leaving the house for ice cream and fresh air. Another describes a first date after repeated failure and thanks the subreddit for making them feel *“a little less lonely and much more hopeful for the future.”* These posts show that strangers in the forum can provide recognition that matters.

The quality of some replies reinforces this. Responding to a post about a humiliating speed dating experience, one commenter writes, *“I just wanted to make sure your post didn’t get ignored.”* That sentence captures something central about the subreddit’s supportive function. Sometimes what is offered is not advice, but witness. Not being ignored becomes meaningful in itself.

At the same time, the same openness that allows support also leaves users exposed. One commenter describes disclosing suicidal thoughts and receiving replies from people trying to buy their PlayStation cheaply because they assumed they would be dead. Another writes, *“There’s people who are actually lonely here and then there’s predators.”* A further comment shows how the presence of hostile or exploitative users affects even well-intentioned participation, with one male user explaining that he is extra cautious because he does not want to be read as incel-like or predatory. In another case, a single sentence, *“I’ve been DMing him and he’s stopped responding,”* captures the quieter form of abandonment that can also define the space.

Taken together, these accounts suggest that the subreddit is best understood as a contested support space. Care is real, sometimes profound, and occasionally transformative. But it is also unstable, and shaped by who posts, who responds, and who is watching for reasons other than solidarity.

#### 5.2.5 Theme 5: rejection of misrecognizing advice

A recurring pattern in the dataset is the rejection of familiar advice scripts. Posts such as *“Love yourself is complete bullshit,” “Never tell a lonely person that there is someone out there for you,”* and *“Just go out and meet people”* do more than express irritation. They challenge the assumption that loneliness can be resolved through a simple change in attitude, greater effort, or a generic phrase.

What users object to is not only poor advice, but the misunderstanding that poor advice represents. A father describing his fifteen-year-old son’s loneliness recounts that the boy stopped him mid-advice and insisted he had *“tried it all.”* The point is not that advice is unwelcome in principle, but that it often comes after effort has already failed. The father’s eventual response, simply holding his son while he cried, quietly recognizes that the standard script had nothing useful left to offer.

This frustration is developed most clearly in posts that dismantle common advice point by point. One user challenges the instruction to *“just go out and meet people”* by walking through parks, cinemas, gyms, and bars, showing why each setting is hostile to connection for someone who is already alone. Another rejects the phrase *“there is someone out there for you,”* calling it poisonous because it repeatedly sets people up for disappointment. In both cases, the complaint is not that hope is always wrong, but that these responses ignore the actual social conditions users are describing.

The same point appears in more indirect ways. A post about turning to an AI companion app after repeated disappointment suggests what happens when conventional social advice stops feeling credible. Another notes that every attempt to post in the subreddit leads to messages from OnlyFans accounts, turning even the support space into a site of commercial exploitation. Across these accounts, simplified advice is experienced not merely as unhelpful, but as a form of misrecognition. It fails because it assumes a much simpler problem than the one users believe they are living through.

#### 5.2.6 Cross-cutting pattern: a gap between what is needed and what is available

Across the five themes, a single structural tension recurs. Users describe not only what they are experiencing, but what they are not receiving. Much of the emotional force of the dataset lies in that gap.

In Theme 1, the gap appears between the need for meaningful connection and the very limited forms of acknowledgment available. In Theme 2, it appears between sustained effort and repeated disappointment. In Theme 3, it appears between the wish to have one’s loneliness recognized and the gendered assumptions that distort that recognition. In Theme 4, it appears between the need for support and the uneven reality of public peer response. In Theme 5, it appears between the complexity of loneliness and the simplicity of the advice often offered in return.

This works best as a thread running through the analysis rather than as a separate theme. It helps explain why frustration, despair, and the search for validation recur so strongly in the dataset. Users are not simply reporting painful experiences. They are reporting painful experiences in contexts where those experiences are often misunderstood, minimized, or met with inadequate responses. The gap, then, is not only between loneliness and connection. It is between what people need in order to feel recognized and what the social world, including the subreddit itself, is actually able to offer.

### 5.3 Mixed-methods integration

The quantitative and qualitative strands addressed related but distinct questions. The quantitative analyses characterized the broader comment corpus by identifying its overall sentiment profile, emotional distribution, recurring lexical clusters, and central keyword relationships. The qualitative analysis examined how loneliness was described, how users interpreted its causes and effects, and how these disclosures were handled within the subreddit. Read together (Table 5), the two strands provide a fuller account than either could offer alone. The quantitative findings show the broad pattern at scale, while the qualitative themes explain how that pattern is produced, lived, and negotiated within specific threads.

**TABLE 5:** Mixed-methods integration of quantitative and qualitative findings.

Area	Quantitative finding	Qualitative interpretation	Integrated insight
Overall emotional tone	Positive sentiment was the largest category (55.44%), with negative (24.40%) and neutral (20.16%) also substantial.	Users frequently disclosed pain, but replies often involved encouragement, validation, and care.	Distress and support coexisted in the same space; the positive signal reflects response patterns, not an absence of suffering.
Emotion profile	Positive emotion had the widest comment reach; trust, joy, and anticipation prominent; negative emotion, sadness, and fear substantial.	Users brought rejection, despair, and low self-worth; replies often offered reassurance and recognition.	Supportive and distress-related language appeared throughout the corpus rather than in separate zones.
Recurring discussion areas	Topic modeling identified gratitude, sympathy, birthday wishes, offers to talk, and romantic or social difficulty.	Loneliness was narrated through milestones, relational disappointment, and the search for acknowledgment.	Recurring lexical clusters reflected the concrete social settings in which loneliness was experienced and responded to.
Central social concerns	Keyword network identified relationship, work, happy, birthday, social, dating, family, and loneliness as central terms.	Themes linked loneliness to intimate relationships, work routine, family context, social exclusion, and personal milestones.	Loneliness was embedded in everyday life rather than framed as an isolated internal state.
Support quality	Computational analyses indicated a broadly supportive emotional environment but could not assess quality or consistency.	Theme 4 showed support was real but uneven, and coexisted with dismissal, tension, and predatory behavior.	The subreddit functioned as a support space, but not a stable or uniformly safe one.
Gender and contestation	Gender was not a dominant computational category; relational and social terms were prominent in lexical analyses.	Theme 3 showed gender structured how loneliness was recognized, dismissed, and argued over.	The qualitative strand surfaced a significant layer of meaning not visible in the aggregate outputs.
Advice and misrecognition	Supportive-sounding language was common computationally, but could not be distinguished from unhelpful advice in context.	Theme 5 showed users rejected conventional advice as simplifying or misreading their situation.	Not all positively coded language was experienced as support; qualitative analysis clarified what computational methods could not.

**TABLE 6:** Forms of loneliness identified in Theme 1.

Form	Description	Illustrative quotation	Theoretical anchor
Social loneliness	Absence of a satisfying peer network; feeling invisible in everyday social settings	“Pure agony, soul crushing. No friends. Sometimes I pretend someone texted me, but it’s really spam. I belong to some WhatsApp groups and I just watch everybody else interacting. Nobody talks to me.”	Weiss (1973): social loneliness
Emotional loneliness	Social contact present but genuine closeness absent; ordinary warmth becomes disproportionately significant	“Does anyone else fall in love with anyone who gives you attention?”	Weiss (1973): emotional loneliness
Existential loneliness	Chronic inner emptiness experienced as a fundamental feature of the self rather than a situational condition	”It’s not about being lonely, it’s all about feeling empty chronically. The feeling of being empty despite doing everything to feel something. Is this apathy? Am I just numb because I’ve been lonely since I ever existed on this planet?”	Van Tilburg (2021): existential loneliness
Bodily loneliness	Absence of physical contact experienced as a distinct deprivation; incidental touch carries unusual emotional weight	“I had a female coworker give me a hug the other day at work and it felt surreal. I have been alone for such a long time that it caught me off guard. It just felt nice. I felt like I was human again for a moment.”	Weiss (1973): social provision through physical contact
Milestone loneliness	Loneliness made acute by socially significant occasions when connection is expected but absent	“Today is my birthday. No one remembered. Not a single person.”	Baumeister and Leary (1995): belonging need intensified at moments of social expectation
Calibrated loneliness	Prolonged absence recalibrates what registers as meaningful contact; minor gestures carry outsized emotional significance	“I’m so lonely that I smile after I see someone upvote me.”	Hawkley and Cacioppo (2010): hypervigilance and heightened sensitivity under chronic loneliness

Quotations are drawn from the qualitative subsample of 80 high-engagement posts and 478 associated comments from r/lonely. Minor spelling corrections have been applied to quotations for readability. Theoretical anchors indicate the primary conceptual lens through which each form is interpreted in relation to the framework presented in section 3.

A first point of convergence concerns the emotional tone of the subreddit. Sentiment analysis showed that the comment corpus leaned positive overall, with 55.44% of comments classified as positive, compared with 24.40% negative and 20.16% neutral. Emotion analysis pointed in the same general direction. Positive emotion had the widest reach across comments, while trust, joy, and anticipation were also prominent. At the same time, negative emotion, sadness, and fear remained substantial. This combination is important. The qualitative analysis helps explain why supportive and distress-related language coexist so strongly. Users often arrived in the subreddit carrying disappointment, rejection, emotional pain, and low self-worth, but the comment space frequently responded with sympathy, encouragement, validation, and attempts at care. In that sense, the positive signal in the quantitative data does not suggest that the subreddit is free of suffering. It suggests that suffering is often met with supportive reply.

The topic and keyword analyses extend this interpretation. Topic modeling identified clusters organized around gratitude, sympathy, encouragement, birthday wishes, offers to talk, and general social or romantic difficulty. The keyword network showed that central terms included relationship, work, happy, understand, birthday, social, dating, experience, problem, loneliness, and family. These outputs suggest that loneliness in this corpus was not discussed as an abstract psychological state. It was tied to intimate relationships, family life, work, social interaction, personal milestones, and the everyday search for recognition. The qualitative themes give those clusters clearer meaning. Theme 1 showed that loneliness took several distinct forms. Theme 2 showed that it was often narrated as the outcome of repeated relational failure. Theme 4 showed that the subreddit could function as a support space, though unevenly. Taken together, the two strands suggest that the corpus is organized not only by pain, but also by attempts to interpret, answer, and manage that pain in public.

The integration is also useful where the strands do not align perfectly. Quantitative methods showed a broadly supportive emotional profile, but they could not on their own explain the recurring tension, hostility, or mistrust visible in particular threads. The qualitative analysis brought those dynamics into view by showing how gendered dismissal, predatory responses, and frustration with platitudinous advice shaped the experience of using the subreddit. In this respect, the qualitative strand complicates the quantitative picture. The subreddit appears supportive in aggregate, but support is uneven, contested, and sometimes undermined by the very response environment in which disclosures occur. This is especially clear in themes concerning gender and the limits of peer support, where users described both recognition and harm within the same space.

The value of integration in this study lies in showing both the scale of the pattern and the specific meanings through which that pattern was produced. The computational analyses established what was prominent at the level of the corpus; the qualitative analysis explained why, in what form, and with what consequence.

## 6 Discussion

### 6.1 Overview of main findings

This study examined loneliness-related discourse in r/lonely through a convergent mixed-methods design that combined large-scale computational analysis with reflexive thematic analysis of a high-engagement qualitative subsample. Three main findings emerged. The different forms of loneliness identified in Theme 1 are summarised in Table 6. First, loneliness in this corpus was clearly multidimensional. Users described social, emotional, existential, bodily, and milestone-based loneliness, and these forms implied different absences and different needs. Second, loneliness was often narrated as the cumulative outcome of relational injury. Rejection, ghosting, betrayal, and repeated disappointment shaped how users understood themselves, others, and the possibility of future connection. Third, the subreddit functioned as a partial and uneven support space. Supportive replies were common, but they coexisted with dismissal, predatory behavior, gendered conflict, and advice that many users experienced as misrecognizing their situation.

Taken together, these findings suggest that loneliness in this setting is best understood not only as disconnection, but as a recurring gap between what users need and what is available to them. That gap appeared in absent or unstable relationships, in the effects of repeated rejection, and in the uneven quality of response offered within the online community itself.

### 6.2 Loneliness as multidimensional

The finding that loneliness took several distinct forms is consistent with Weiss’s (1973) typology and with more recent multidimensional approaches that treat loneliness as heterogeneous rather than unitary (Van Tilburg, 2021; Walsh et al., 2025). What this study adds is a view of that plurality in naturally occurring peer discourse. Posts concerning touch starvation, birthday loneliness, romantic absence, friendlessness, and existential isolation appeared alongside one another in the same community, yet they pointed to different deficits in connection and different forms of pain.

The data also suggest that prolonged loneliness can recalibrate what counts as meaningful contact. Brief gestures such as an upvote, a short message, or a moment of physical contact sometimes carried unusual emotional weight because more substantial connection was absent. This is consistent with Hawkley and Cacioppo’s (2010) account of the loneliness regulatory loop, in which perceived social isolation triggers hypervigilance for threat, producing cognitive biases that deepen withdrawal over time. What the present data add is a narrative dimension to that mechanism: users do not simply withdraw silently—they articulate the process publicly, describing the moment when ordinary acknowledgment became unusually significant. This extends Hawkley and Cacioppo’s neurobiological account into the domain of peer discourse, showing how the regulatory loop is expressed and recognized in a social setting.

#### 6.3 Loneliness as social injury

A substantial part of the dataset framed loneliness as the result of repeated relational harm rather than mere absence. Users wrote about ghosting, betrayal, exclusion, and sustained failure in dating or friendship in ways that made loneliness appear cumulative. These were not isolated disappointments. They were narrated as experiences that had altered expectations, reduced willingness to reach out, and reshaped beliefs about whether connection was available at all.

This pattern aligns with research showing that rejection and exclusion are experienced as forms of social pain (Cacioppo and Cacioppo, 2014) and with work on the particular injury caused by ghosting (Pancani et al., 2022). The Reddit material extends that literature by showing how users narrate this process over time. This extends the existing literature in one specific way. Cacioppo and Cacioppo (2014) and Pancani et al. (2022) document the psychological mechanisms of rejection and ghosting in controlled or experimental settings. The present data show how those mechanisms accumulate and are narrated over time in unsolicited peer discourse, producing settled interpretive frames (not just transient emotional responses) about the self and the social world. That longitudinal and narrative quality is not captured in experimental designs and represents a genuine addition to what is currently known.

### 6.4 The subreddit as a partial and uneven support space

The computational strand showed that positive sentiment was the largest category in the comment corpus, and the emotion analysis similarly indicated strong positive and trust-related language at scale. On its own, that might suggest that r/lonely functioned as a broadly supportive space. The qualitative findings complicate that picture. Support was real and, in some cases, clearly meaningful. Yet the same forum also contained objectifying responses, predatory contact, internal conflict, and advice experienced as superficial or invalidating.

This unevenness is important. The positive aggregate profile does not mean the subreddit was uniformly supportive. It means that supportive-sounding language was common in the corpus. The mixed-methods design was especially useful here. The computational analyses established the broader tone of the corpus, while the thematic analysis showed that the same response environment could contain care, tension, misrecognition, and harm.

### 6.5 Gender, recognition, and misrecognition

Gender was one of the most analytically significant dimensions of this study, shaping both how loneliness was described and how disclosures were received by others in the community. Across the qualitative material, loneliness claims were sometimes validated, but they were also dismissed, reframed, sexualized, or met with advice that users felt missed the point. This was especially visible in gender-related threads. Male users often described their loneliness being recast as sexual frustration or weakness, while female users described it being dismissed on the assumption that male (sexual) attention should be sufficient to prevent it.

Honneth’s (1996) account of recognition provides a useful framework here. In this perspective, harm does not arise only from missing relationships, but also from being denied acknowledgment as someone whose needs and self-understanding are legitimate. This extends Honneth’s (1996) framework beyond its original institutional and interpersonal contexts into a digital peer community where recognition is distributed publicly, anonymously, and unevenly. Prior Reddit research has examined self-disclosure and anonymity (De Choudhury and De, 2014) and the causes and intensities of loneliness (Salzmann-Erikson and Olsson, 2025), but neither examined how the response environment itself (who validates, who dismisses, who exploits) shapes the experience of loneliness within the platform. That is the specific contribution of this study to the literature on digital peer support.

### 6.6 What the mixed-methods design adds

The mixed-methods design was analytically necessary because neither strand alone could have produced an adequate account of the subreddit. The computational analyses established the broad emotional and lexical structure of the corpus, showing that supportive and positive language was common and that key areas of discussion included relationships, work, family, social life, and personally meaningful occasions.

The qualitative strand added what those aggregate findings could not provide. It showed what kinds of interactions sat behind the general patterns, how users narrated loneliness in relation to rejection and recognition, and how replies could both alleviate and worsen the experience being disclosed. This is an important methodological point for research on sensitive online discourse. Aggregate textual patterns are valuable, but they remain incomplete without close interpretive analysis.

### 6.7 Limitations

Several limitations should shape interpretation of the findings. First, the study is based on a single public subreddit. r/lonely has its own norms, audience, and interactional culture, and these may not reflect other online spaces or offline experiences of loneliness. Reddit’s user base is also uneven and tends to be younger, Western, and male-skewed, which likely shapes who is visible in the dataset and how loneliness is discussed (Amaya et al., 2021; Proferes et al., 2021). The findings should not be generalized beyond this specific community and dataset. They describe how loneliness was expressed and responded to in r/lonely during the period studied, and cannot be taken as representative of how loneliness is experienced or discussed in other online comunities, offline contexts, or the broader population.

Second, the qualitative sample was drawn from the 80 most liked posts and their associated comment threads. This made close analysis feasible and ensured that the sample captured highly visible and highly engaged discussions, but it also means that quieter disclosures and low-engagement interactions are less visible in the findings. Experiences that did not attract community engagement (including those of users from minority backgrounds, users who posted once without response, or users whose disclosures were less publicly resonant) are not well represented in the qualitative findings. This is an important constraint on the scope of the interpretive claims made in this study.

Third, the computational analyses have known limitations, including difficulty detecting sarcasm, irony, and context-dependent meaning. Topic modeling and keyword network analysis were conducted on large but capped subsets of the corpus rather than the full dataset. These analyses were used as exploratory descriptive tools to map broad lexical structure and should not be read as statistically generalizable estimates of full-corpus patterns. The primary corpus-level quantitative findings rest on the sentiment and emotion analyses, which were conducted on the complete set of 877,799 comments. Finally, the study analyzed how loneliness was expressed and responded to in this digital setting. It cannot determine users’ offline circumstances or longer-term outcomes. Taken together, these methodological constraints mean that the study’s claims are bounded: they describe patterns within a specific digital setting, analyzed through methods with known interpretive limits, and interpreted through a qualitative lens that reflects the positionality of the researchers involved.

### 6.8 Implications

The findings have implications for loneliness research, digital peer support, and methods. For loneliness research, they support a multidimensional view of the phenomenon and suggest that studies treating loneliness as a single construct may obscure important variation in what is missing and how that absence is experienced. The distinction between loneliness as absence and loneliness as accumulated relational injury is especially important.

For digital peer support, the findings suggest that the supportive character of an online forum cannot be inferred from overall sentiment or the volume of replies alone. Platform structure, moderation, and community norms matter. Public forums may provide recognition and relief for some users while exposing others to dismissal, predation, or intensified distress. For research design, the study shows the value of combining computational and qualitative approaches when analyzing sensitive online discourse. Aggregate measures can identify scale and pattern, but they cannot by themselves explain how interaction works.

## 7 Conclusion

This study examined how loneliness is described, experienced, and responded to in r/lonely, one of the largest public loneliness communities on Reddit. The findings show that loneliness in this setting is multidimensional, frequently shaped by accumulated relational injury, and negotiated within a response environment that offers both support and harm. Users did not only describe missing connection. They also described being misrecognized when they tried to name that loss, and the subreddit itself was part of that dynamic rather than standing outside it.

More broadly, the findings suggest that online loneliness forums may provide important spaces of disclosure and recognition, but they do not remove the social conditions that produce loneliness, nor do they guarantee that vulnerable disclosures will be met with care.

## Data Availability

Publicly available datasets were analyzed in this study. This data can be found at: https://www.reddit.com/r/lonely/.
